# Different Stability and Proteasome-Mediated Degradation Rate of SMN Protein Isoforms

**DOI:** 10.1371/journal.pone.0134163

**Published:** 2015-07-27

**Authors:** Denise Locatelli, Mineko Terao, Mami Kurosaki, Maria Clara Zanellati, Daniela Rita Pletto, Adele Finardi, Francesca Colciaghi, Enrico Garattini, Giorgio Stefano Battaglia

**Affiliations:** 1 Molecular Neuroanatomy and Pathogenesis Unit, IRCCS Neurological Institute “C. Besta”, Milano, Italy; 2 Laboratory of Molecular Biology, IRCCS-Istituto di Ricerche Farmacologiche "Mario Negri", Milano, Italy; University of Edinburgh, UNITED KINGDOM

## Abstract

The key pathogenic steps leading to spinal muscular atrophy (SMA), a genetic disease characterized by selective motor neuron degeneration, are not fully clarified. The full-length SMN protein (FL-SMN), the main protein product of the disease gene *SMN1*, plays an established role in the cytoplasm in snRNP biogenesis ultimately leading to mRNA splicing within the nucleus. It is also involved in the mRNA axonal transport. However, to what extent the impairment of these two SMN functions contributes to SMA pathogenesis remains unknown. A shorter SMN isoform, axonal-SMN or a-SMN, with more specific axonal localization, has been discovered, but whether it might act in concert with FL-SMN in SMA pathogenesis is not known. As a first step in defining common or divergent intracellular roles of FL-SMN vs a-SMN proteins, we here characterized the turn-over of both proteins and investigated which pathway contributed to a-SMN degradation. We performed real time western blot and confocal immunofluorescence analysis in easily controllable in vitro settings. We analyzed co-transfected NSC34 and HeLa cells and cell clones stably expressing both a-SMN and FL-SMN proteins after specific blocking of transcript or protein synthesis and inhibition of known intracellular degradation pathways. Our data indicated that whereas the stability of both FL-SMN and a-SMN transcripts was comparable, the a-SMN protein was characterized by a much shorter half-life than FL-SMN. In addition, as already demonstrated for FL-SMN, the Ub/proteasome pathway played a major role in the a-SMN protein degradation. We hypothesize that the faster degradation rate of a-SMN vs FL-SMN is related to the protection provided by the protein complex in which FL-SMN is assembled. The diverse a-SMN vs FL-SMN C-terminus may dictate different protein interactions and complex formation explaining the different localization and role in the neuronal compartment, and the lower expression and stability of a-SMN.

## Introduction

Spinal Muscular Atrophy or SMA is a severe autosomal recessive disease characterized by selective motor neuron degeneration. SMA is the leading genetic cause of infant mortality, with an incidence of 1 in 6,000–10,000 neonates, a prevalence of 1 in 53,000 individuals, and an estimated carrier frequency of 1 in 40 [1–3]. SMA is classified into three main clinical types (I-III), in relation to the age of onset and disease severity. In affected children, motor neuron loss leads to progressive amyotrophic paralysis, respiratory failure, and, in more severe cases, to death.

SMA is genetically determined by disruptions of the Survival of Motor Neuron 1 (*SMN1*) gene, first reported in 1995 [4]. The different disease severity of affected patients is related to the presence, peculiar to the human species, of the almost identical *SMN2* copy gene. In contrast to *SMN1*, which produces the functional “full-length” FL-SMN protein, the *SMN2* gene mainly encodes an exon 7 truncated SMN form (Δ7-SMN), unstable and rapidly degraded, and only low amounts of FL-SMN [5–10]. Thus, the presence of multiple *SMN2* copies, although not preventing the disease expression, may ameliorate the clinical course of SMA [11,12]. An additional SMN form has been also identified (axonal-SMN or a-SMN) [13]. The a-SMN protein is mainly produced by the *SMN1* gene through an intron-retention event [13]. In comparison to FL-SMN, the a-SMN protein is more selectively expressed in axons and stimulates axon growth when over-expressed in vitro [13,14].

Regarding function, a number of studies have clearly demonstrated that FL-SMN is part of a macromolecular complex playing a fundamental role in spliceosomal biogenesis and mRNA splicing [15–20]. However, it is not as yet clear whether impairment of splicing is the key pathogenic step leading to SMA [21–24]. FL-SMN has been localized in axons and growth cones of developing motor neurons [25–27], and several studies have suggested a role for FL-SMN in the axonal transport of mRNAs [28–31]. Thus, the loss of this specific function might lead to the motor neuron failure typical of SMA [28].

While FL-SMN functions are still highly debated, the a-SMN role in vivo is even more uncertain. First, the cell mechanisms set in motion by a-SMN are not clarified. Second, the link between a-SMN and SMA is uncertain [32], even if the disruption of the a-SMN axonogenic properties by SMA mutations might suggest a role in SMA pathogenesis [14]. Finally, it is not as yet clear whether a-SMN might act in concert with FL-SMN, even if the potential mediators of a-SMN biological activity in axon growth and cell motility, i.e., the CCL2 and CCL7 chemokines and the growth factor IGF1, might indicate a cell role of a-SMN distinct from that of FL-SMN [33].

A relevant difference between a-SMN and FL-SMN is the protein amount within the cell. In contrast to FL-SMN, the a-SMN protein is detectable by Western blot only during development [13]. Once development is completed, a-SMN becomes almost undetectable in most cell types as well as in neuronal and non-neuronal tissues. As a first step to define common or divergent intracellular roles of FL-SMN vs a-SMN proteins, in the present paper we characterized the turn-over of a-SMN vs FL-SMN proteins and investigated which pathway contributed to a-SMN degradation.

## Materials and Methods

### Cell culture

NSC34 motor neurons [34] were routinely maintained in Dulbecco's modified Eagle's medium (DMEM) supplemented with 5% fetal bovine serum, 1mM glutamine and antibiotics (penicillin G K-salt, 100UI/ml and streptomycin sulphate, 100 μg/ml) and grown at 37°C in a humidified atmosphere (5% CO_2_−95% air) in 25 cm^2^ flasks (Corning, Cambridge, MA, USA). Every week cells were detached from the plates by mechanical dissociation in culture medium, and then replated at a density of 5 x 10^4^ cells/flask. The a-SMN expressing clones [33] were cultured in low-glucose (1 μg/l) DMEM medium (Life Technologies, Carlsbad, CA) supplemented with 5% TET-System-approved fetal bovine serum (Clontech, Mountain View, CA) in the presence of 10 μg /ml of Blasticidin S and 50 μg /ml Zeocin (Life Technologies). For Western blot (WB) and immunofluorescence (IF) experiments, the cells were grown in culture dishes pre-coated for one hour with Matrigel Matrix Basement Membrane (BD Bioscience, Bedford, MA) diluted 50 times in DMEM.

### Plasmid generation and transfections

Human FL-SMN and a-SMN cDNA fragments were in frame cloned in the pcDNA4/HisMaxTOPO expression vector (Life Technologies) as previously reported [13]. All clones were fully sequenced. Transfection was performed with Lipofectamine Plus (Life Technologies) by standard procedures. For WB analysis, NSC34 cells were washed twice in ice-cold phosphate-buffered saline (PBS) without Ca^++^ and Mg^++^ ions, detached with a cell scraper in 2 ml of the same buffer, and centrifuged at 1,000g for 5 min.

### Protein stability assay

HeLa and NSC34 cells were co-transfected with equal amount of pcDNA4/a-SMN and pcDNA4/FL-SMN. After 20 hrs cells were treated with 100 μg/ml cycloheximide (CHX, Calbiochem, Darmstadt, Germany) to inhibit protein synthesis. Cycloheximide-treated cells were harvested at different time points (0, 1, 3, 5 and 7 hrs) and processed for immunoblotting with anti-tag antibody (anti-Xpress, Life Technologies). Anti-actin antibody was used as internal controls. To evaluate the effect of ubiquitin proteasome pathway (UPP) block on a-SMN and FL-SMN half-life, NSC34 cells were co-transfected as reported above and after 20 hrs were treated with MG132 (5 μM: Sigma-Aldrich, St. Louis, MO, USA). At 40 hrs post transfection, cells were treated with 100 μg/ml CHX, harvested at the time points considered (1,3,5,7 hrs) and processed for WB analysis.

### Antibodies

The anti-peptide polyclonal antibody raised against the C-terminal region of the human a-SMN (#910) was prepared in rabbits in 2005 by NeoMPS (Strasbourg, France; diluted 1:500 for IF). The specific antibody samples used in this study have been previously described [13]. Mouse anti-tag (anti-Xpress), revealing transfected proteins only, was purchased from Life Technologies (diluted 1:500 for WB and IF experiments), polyclonal anti-Flag from Sigma-Aldrich (diluted 1:500 for IF); anti-SMN clone 8 from BD Transduction Laboratories (diluted 1:20,000 for WB); mouse anti-actin from Millipore (diluted 1:5,000 for WB); mouse anti-neurofilament 200 (NF-200) from Sigma-Aldrich (diluited 1:500 for IF).

### Western blot analysis

Transfected cell cultures were lysed in buffer containing 0.1M Na-phosphate (pH 7.4, 0.2% Triton X-100, 0.1 mM EDTA, 0.2 mM PMSF, 1 μg /ml aprotinin and 1 μg /ml leupeptin) by three-repeated freezing and thawing cycles. The resulting cell lysates were centrifuged at 13,000 g for 10 min. Proteins were separated by SDS-PAGE (12% acrylamide) and electro-blotted on nitrocellulose paper for 1 hrs at 180mA. The nitrocellulose was blocked overnight with 10% no-fat milk in Tris buffered saline (TBS). The primary antibodies were diluted in 5% no-fat milk in TBS and incubated with the nitrocellulose for 1.5 hrs. The membranes were rinsed in TBS-tween 20, and incubated with the secondary antibodies (IRDye 800-labeled goat-anti- mouse IgG, LI-COR Biosciences, Lincoln, NE; diluted 1:15,000) in 5% non fat milk in TBS for 45 min. The Odyssey infrared imaging system (LI-COR Biosciences) was used to measure protein concentration. Scanning parameters (non-saturated signals, resolution of 84 or 169 μm for high/medium quality) were set according to the manufacture’s instructions. Outlines were drawn around the bands and the integrated intensity was calculated after subtracting background. The amount of SMN protein was normalized versus actin signals and compared among groups.

### Quantitative analysis of FL- and a-SMN transcripts

NSC34 cells were co-transfected with human FL-SMN and a-SMN cDNAs and subsequently treated with actinomycin D (actD, Sigma-Aldrich; 5 μM, inhibitor of transcription) or CHX (100 μg/ml, inhibitor of translation). The total RNA, prepared using miRNeasy Mini Kit (Qiagen, Valencia, CA), was reverse-transcribed, according to the standard procedure using GeneAmp RNA PCR Core Kit (Applied Biosystems, Branchburg, NJ). The levels of FL-SMN and a-SMN transcripts were determined by quantitative RT-PCR (qRT-PCR) with SYBR green (Applied Biosystems), using oligonucleotides amplimers specific for human FL-SMN (5’-ctgtgttgtggtttacactgg-3’, the nucleotides 529–549 and 5’-cactttcatctgttgaaacttggc-3’ complementary to the nucleotides 650–673 of the human SMN sequence NM_00034), and specific for human a-SMN (5’-aaatctgtccgatctactttcc-3’, the nucleotides 571–592 of NM_00034 and 5’-acagtttctcatctagtctctgc-3’, complementary to the nucleotides 38–60 of the third intronic sequence: 17,656–17,678 of the human SMN1 genomic sequence NC_000005.10). None of the amplimers recognized murine endogenous FL-SMN nor a-SMN transcripts. The results were normalized using the Taqman probe specific for mouse beta-actin (Actb, Mm01205647_gl. Applied Biosystems).

### Drug Treatment

To verify a-SMN and FL-SMN processing, NSC34 cells were co-transfected with pcDNA4/a-SMN and pcDNA4/FL-SMN and after 20 hrs were treated with the proteasome inhibitors MG132 (5 μM) or lactacystin (2.5 μM, Sigma-Aldrich) or a calpain inhibitor calpeptin (50 μM, Calbiochem, Darmstadt, Germany). Treated cells were harvested at different time points (8, 16 hrs). Similar experiments were also performed on the a-SMN81 clone. After 24hrs plating, cells were treated with MG132 (5 μM) or lactacystin (2.5 μM) for 8 or 16 hrs. For WB analysis, treated cells were harvested and pellets were frozen at -80°C until use.

### Immunofluorescence analysis

Transfected cells were fixed with 4% paraformaldehyde in 4% sucrose in phosphate buffer pH. 7.2 (PB). To avoid a crosslink with non-specific epitopes, cells were rinsed three times in low concentration salt buffer (LS; 150 mM NaCl and 10 mM PB at pH 7.4) and three times in high concentration salt buffer (HS; 500 mM NaCl and 20 mM PB at pH 7.4), and then incubated in goat serum dilution buffer (GSDB, 3% normal goat serum, 0.1% Triton X-100, 500 mM NaCl and 20 mM PB at pH 7.4). Cells were incubated with primary antibodies for 3hrs at room temperature and then for 1hrs with Alexa Fluor 488 or 546 antibodies (Molecular Probes, Eugene, OR, USA; diluted 1:2,000). Cells were repeatedly rinsed, coverslipped with Fluorsave (Calbiochem) and examined on a TCS SP8 confocal microscope (Leica Microsystems, Wetzlar, Germany).

### Cell measurements

Confocal microscope images (200x magnification) were captured from three different experiments in each setting (in both co-transfected NSC34 and a-SMN cell clones, with or w/o MG132). At least 50 neurons completed included in the imaged fields were randomly selected for each group. Axon length, somatic area, axonal abnormalities (i.e., irregular neurites with or without neuritic swellings), and FL-SMN distribution were quantified by means the Freehand Line Tool of the Image-ProPlus software.

### Statistical evaluation

The significance of the difference in a-SMN *vs* FL-SMN mRNA expression, and the effect of MG132 on FL-SMN and a-SMN half-life were evaluated by unpaired Student’s t-test. Differences in mean axon length and somatic area between treated and untreated cells were assessed by unpaired Student’s t-test, whereas differences in neuritic abnormalities and FL-SMN distribution were assessed by chi-square test. All the other statistical analyses were performed by means of one-way ANOVA followed by Tukey HSD as post-hoc comparison test, using a free web utility (http://vassarstats.net/). All data were expressed as mean ± SEM of three independent experiments, and difference considered significant when p<0.05.

## Results

### a-SMN has a shorter half-life than FL-SMN

**Fig 1:**
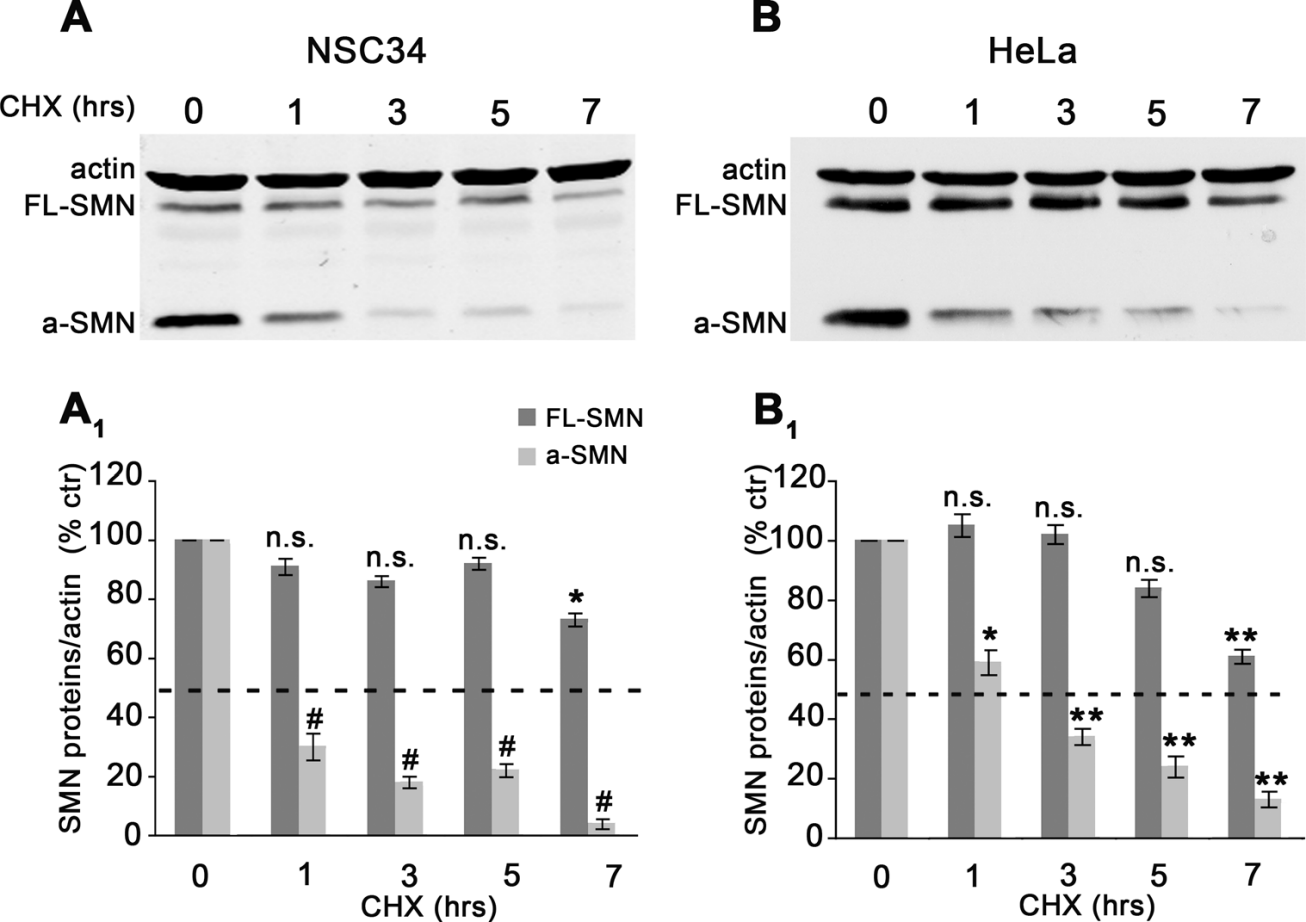
FL-SMN and a-SMN protein half-life. Representative Western blots (**A-B)** and graphs (**A**_**1**_ **- B**_**1**_: OD ratio *vs* actin) of FL-SMN and a-SMN protein expression levels after cycloheximide (CHX) treatment. NSC34 (**A**) and HeLa (**B**) cells were first co-transfected with N-terminally tagged human FL-SMN and a-SMN, then treated with CHX and harvested at different time-points. The a-SMN protein levels were already significantly reduced after 1hrs CHX and almost absent after 7hrs (A_1_- B_1_) whereas the FL-SMN protein levels were significantly reduced after 7 hrs CHX only (A_1_-B_1_). Data in **A**_**1**_ **- B**_**1**_ are presented as mean ± SEM of three different experiments. The differences in protein levels were evaluated by means of one-way ANOVA followed by Tukey HSD as post hoc comparison test in NSC34 (A_1_) and HeLa cells (B_1_) (* p<0.05; **p<0.01; # p<0.001). Distinct one-way ANOVA tests were performed for the two protein datasets, i.e., one for a-SMN and one for FL-SMN. In the graphs, the statistical differences for each time point were reported vs untreated cells (0 hrs). a-SMN in NSC34 cells (A_1_): 1, 3, 5 and 7 hrs vs 0 hrs, #p<0.001; a-SMN in HeLa cells (B_1_): 1 hrs vs 0 hrs, *p<0.05; 3, 5 and 7 hrs vs 0 hrs, **p<0.01; FL-SMN in NSC34 cells (A_1_): 1, 3, 5 vs 0 hrs, n.s.; 7 hrs vs 0 hrs, *p<0.05; FL-SMN in HeLa cells (B_1_): 1, 3, 5 vs 0 hrs, n.s.; 7 hrs vs 0 hrs, **p<0.01.

Relative to FL-SMN, a-SMN is characterized by much lower steady-state levels in spinal motor neurons in vivo [13]. We therefore investigated mechanisms possibly underlying the a-SMN low intra-cellular levels, using easily controllable settings such as cultured cells in vitro. We first compared possible difference in the FL-SMN vs a-SMN intracellular protein stability. To do this, we verified the intra-cellular level of the two proteins after transient co-expression of N-terminally tagged constructs encoding human a-SMN and FL-SMN for 20 hrs, and subsequent block of protein synthesis through cycloheximide (CHX) treatment (Fig 1). NSC34 cells were harvested at different time points (0, 1, 3, 5, 7 hrs) after CHX and the protein levels examined through quantitative western blot analysis. As shown in Fig 1, WB of cell lysates revealed that a-SMN protein levels were already significantly reduced after 1hrs of CHX treatment and almost absent after 7 hrs (Fig 1A and 1A_1_). By contrast, FL-SMN protein levels were significantly reduced after 7 hrs of CHX treatment only (Fig 1A and 1A_1_). To verify different a-SMN vs FL-SMN stability also in non-neuronal cells, we performed similar experiments in the human epithelial cell line HeLa, confirming rapid and progressive reduction of a-SMN after CHX block and much slower reduction of FL-SMN, which again became significant only after 7 hrs of CHX treatment (Fig 1B and 1B_1_).

**Fig 2:**
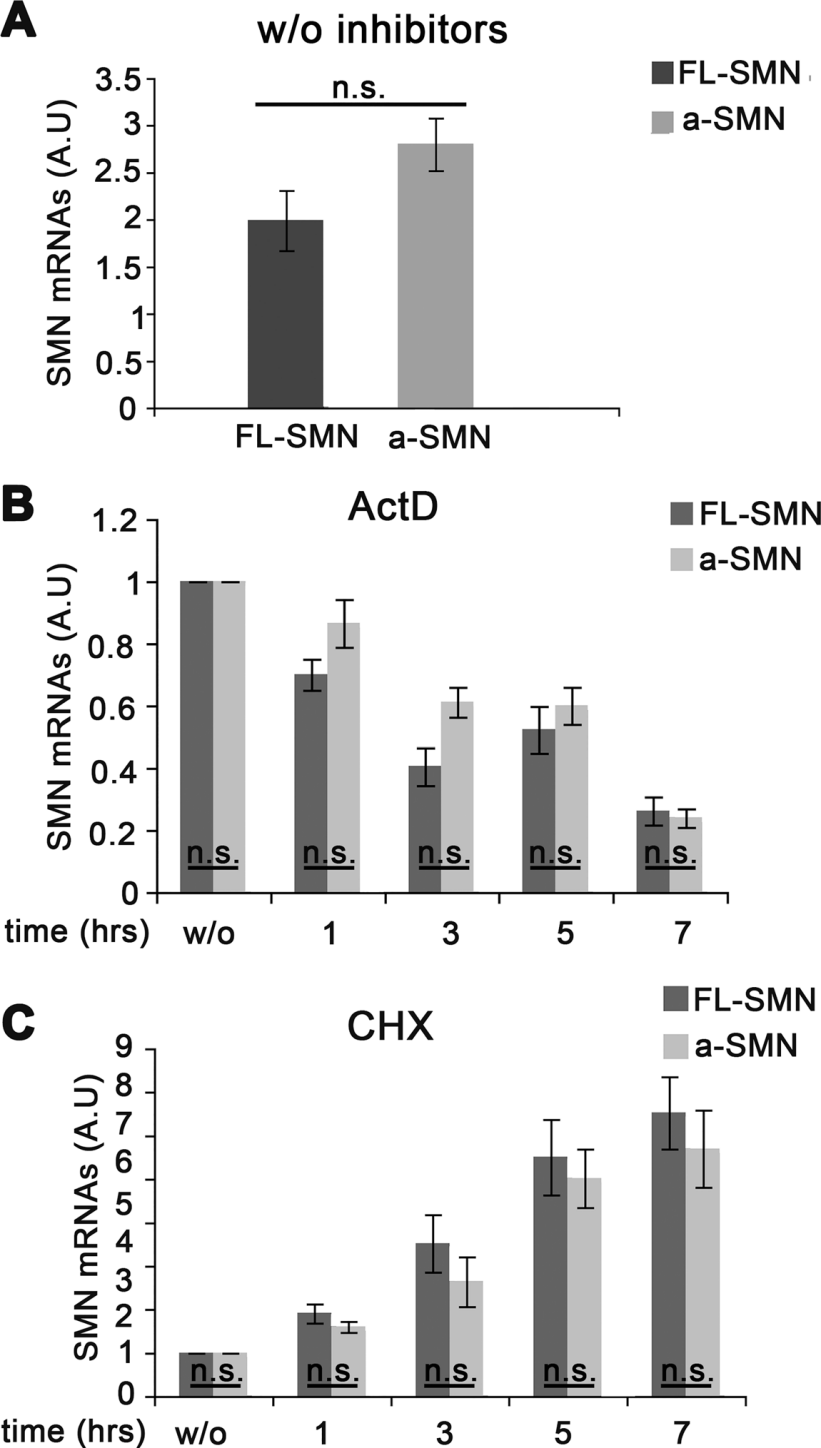
FL-SMN and a-SMN mRNA stability. **A-C**. Quantitative RT-PCR analysis of FL-SMN and a-SMN transcript expression. NSC34 cells were co-transfected with human FL-SMN and a-SMN cDNA, untreated (**A**), or treated with actinomycin D (actD; **B**) or cycloheximide (CHX;**C**). Not significant (n.s.) differences in FL-SMN *vs* a-SMN transcript expression were found at any time-point and in any condition considered (A-C). Data were presented as mean ± SEM of three different experiments. Statistical analysis was performed by Student’s t-test.

We next verified whether the different a-SMN vs FL-SMN protein levels observed in vivo [13] were due to different stabilities of the corresponding mRNAs. NSC34 motor neurons were transiently co-transfected with a-SMN and FL-SMN constructs for 20 hrs and then treated with either actinomycin D, to block transcription, or cycloheximide, to block protein translation and allow mRNA accumulation. After transfection, the basal expression (i.e., before either treatments) of the two mRNAs, as measured by qRT-PCR, did not differ significantly (Fig 2A). After actinomycin D treatment, both a-SMN and FL-SMN levels showed comparable and progressive decrease, and no difference in the mRNA decay became evident (3–5 hrs half-life for FL-SMN and 5 hrs for a-SMN mRNA: Fig 2B). Similar results were obtained after cycloheximide treatment. As shown in Fig 2C, CHX treatment led to the increase of both FL-SMN and a-SMN mRNA levels (approximately by 7-fold at 7 hrs vs untreated cells), and no difference between the two transcripts became evident at any time-point considered after CHX (Fig 2C). These latter data showed that the transcripts levels for both SMN protein isoforms were comparable and no mRNA differences could explain the different intracellular fate of FL-SMN and a-SMN proteins. Taken together, our results demonstrated that a-SMN and FL-SMN were characterized by clearly different protein stability unrelated to mRNA expression, therefore suggesting that the low steady-state a-SMN levels might be controlled by post-translational mechanisms.

### a-SMN half-life is extended by proteasome inhibitors

**Fig 3:**
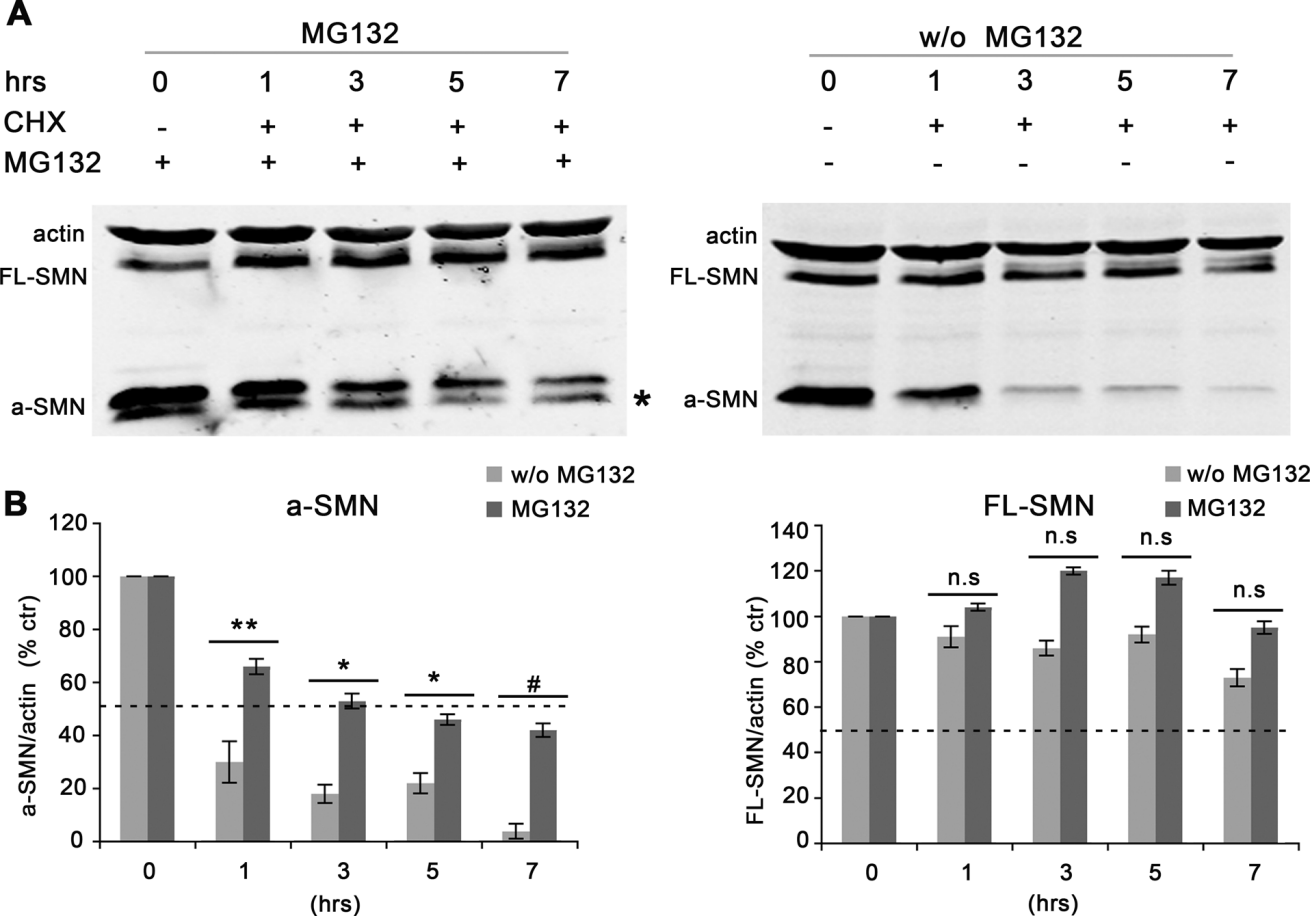
The proteasome inhibitor MG132 extends FL-SMN and a-SMN protein half-life. Representative Western blots (**A)** and bar graphs (**B**: OD ratio vs actin) of FL-SMN and a-SMN protein expression levels after cycloheximide (CHX) treatment in the presence **(A**, left**)** or absence **(A**, right**)** of MG132. The “+” and “-”indications represent the presence or absence, respectively, of MG132 and CHX. NSC34 cells were first co-transfected with N-terminally tagged human FL-SMN and a-SMN, then treated with CHX for 0, 1, 3, 5 and 7 hrs with or without MG132. The MG132 treatment significantly increased the a-SMN half-life at all time-point considered (**B**, left), whereas the MG132 effect on FL-SMN was not statistically significant (**B**, right). Data in **B** are presented as mean ± SEM of three different experiments. For both FL-SMN and a-SMN, distinct statistical analysis was performed by Student’s t-test between groups w/o and with MG132 treatment at each time points considered (n.s. = not significant; *p<0.05; **p<0.01; #p<0.001).

We next verified whether inhibiting the proteasome system could counteract the rapid degradation of a-SMN observed after CHX. NSC34 motor neurons co-transfected with N-terminally tagged a-SMN and FL-SMN constructs were treated with the proteasome inhibitor MG132 for 16hrs or left untreated, and then incubated with CHX. NSC34 cells were harvested at the different time points considered in previous experiments. WB analysis revealed that the MG132 treatment was able to delay significantly the CHX-related degradation of the a-SMN protein by extending the half-life of a-SMN from approximately 1 to 5 hrs (see WB in Fig 3A, and quantification in Fig 3B, left). Notably, in MG132 pre-treated NSC34 cells an additional a-SMN protein band of lower molecular weight (around 22 kDa) became evident (Fig 3A left, asterisk) possibly related to protein cleavage at the C-terminus, since the antibody used recognized the a-SMN N-terminal tag. We confirmed that the 22 kDa band was related to a-SMN since it was present in single transfection experiments after a-SMN but not FL-SMN transfection (data not shown). MG132 treatment also affected FL-SMN degradation by slightly increasing protein levels (Fig 3B, right). The effect however was quantitatively less pronounced than that exerted on a-SMN stability, and it was statistically not significant (Fig 3B, right).

### Proteasome inhibitors increase a-SMN/FL-SMN protein levels in NSC34 cells and a-SMN clones

**Fig 4:**
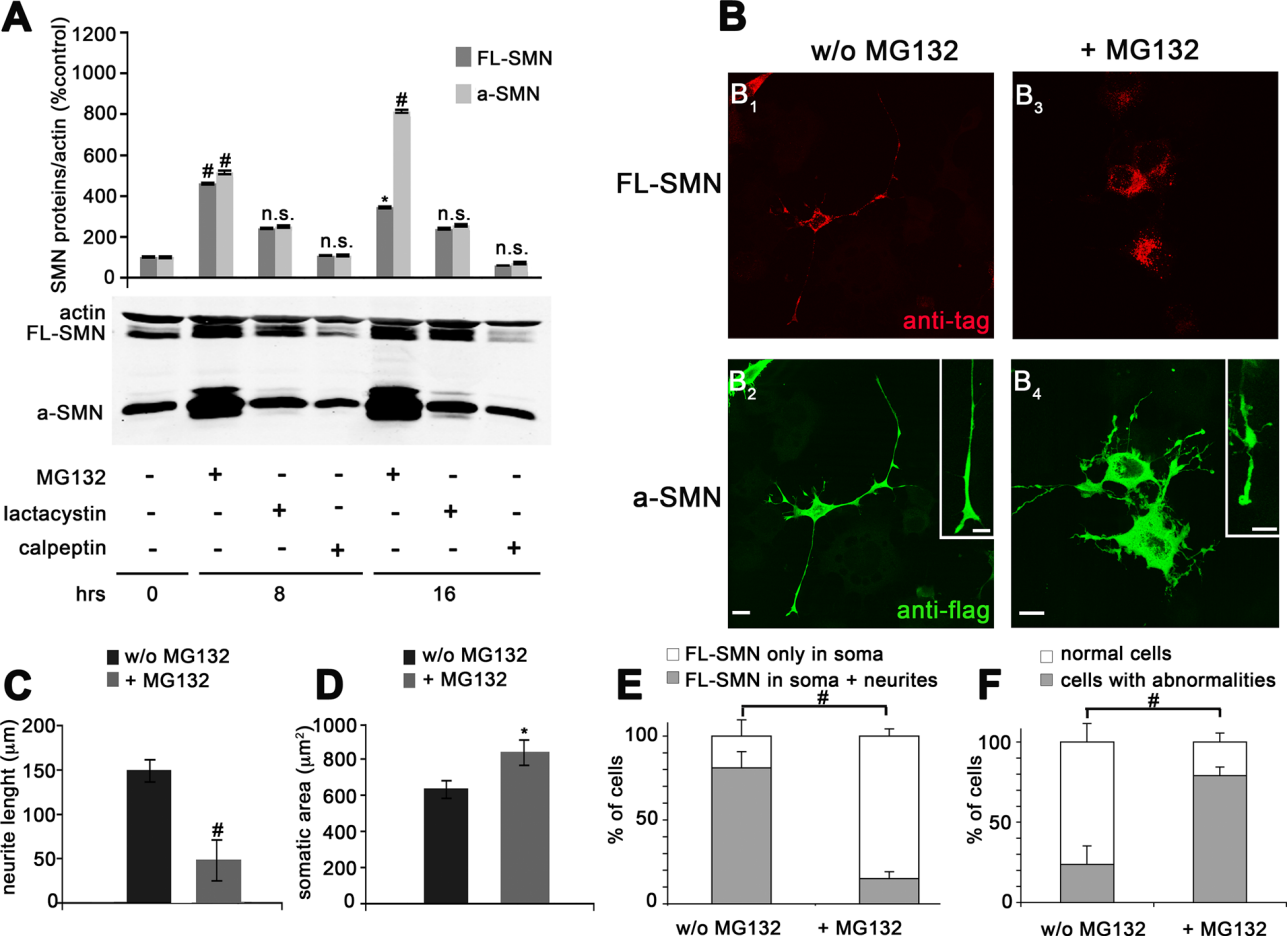
Different effects of proteasome and calpain inhibitors on FL-SMN and a-SMN protein levels in NSC34 motor neurons. **(A)** Western blot analysis and quantification of FL-SMN and a-SMN protein expression after proteasome or calpain inhibition. NSC34 cells were first co-transfected with N-terminally tagged human FL-SMN and a-SMN and then treated with the proteasome inhibitors MG132 or lactacystin, or the calpain inhibitor calpeptin. The “+” and “-”indications represent the presence or absence, respectively, of MG132, lactacystin or calpeptin. The MG132 treatment significantly increased both a-SMN (8 and 16 hrs vs 0 hrs, #p<0.001) and FL-SMN protein levels (8 hrs vs 0 hrs, #p<0.001; 16 hrs vs 0 hrs *p<0.05). Note however that the MG132 effect was progressive over time for a-SMN only. Non-significant differences (n.s.) were detected after either lactacystin or calpain treatment for both proteins vs untreated cells (0 hrs groups). Distinct statistical tests were performed for the two protein datasets, i.e., one for a-SMN and one for FL-SMN. Statistical analysis was performed by one-way ANOVA followed by Tukey HSD as post hoc comparison test. (**B**) Representative confocal IF images illustrating the sub-cellular localization of both SMN isoforms in NSC34 cells in the absence (B_1_- B_2_) or presence (B_3_- B_4_) of MG132. The MG132 treatment induced shorter, thicker, irregular neurites with frequent neuritic swellings (compare insets in B_2_ vs B_4_). FL-SMN was localized in coarser cytoplasmic granules, not extending into neurites (B_3_), whereas a-SMN was diffusely distributed in the cell body and concentrated in the neuritic swellings (B_4_). Scale bars: 35 μm; 20 μm in insets. (**C-D**) Bar graphs showing the quantification of neurite length (C) and soma size (D) of co-transfected cell treated and untreated with MG132. Statistical analysis was performed by Student’s t-test (*p<0.05; #p<0.001). (**E, F**) Stacked histograms showing the FL-SMN distribution (E) and cell abnormalities (F) of co-transfected cell untreated and treated with MG132. Data are presented as mean ± SEM of three different experiments. Percent ratio of FL-SMN distribution (FL-SMN only in soma/ FL-SMN in soma + neurites) and percent ratio of cell abnormalities (normal cells/ cells with abnormalities) were compared by means of chi-square test (#p<0.001) between the two experimental conditions (w/o MG132 or + MG132).

To better define the intracellular mechanisms contributing to a-SMN and FL-SMN protein degradation, co-transfected NSC34 motor neurons were treated with different proteasome inhibitors (MG132 and lactacystin) and the calpain inhibitor calpeptin (Fig 4). Cells were harvested at different time points (0, 8 and 16 hrs) and protein expression analyzed by WB. As shown in Fig 4, MG132 treatment significantly increased both a-SMN and FL-SMN levels at the time-points considered (Fig 4A). However, the effect of MG132 was more evident on a-SMN than FL-SMN protein levels and progressive over time for a-SMN only (Fig 4A). Lactacystin tended to exert similar effect on the intracellular levels of both proteins after 8 and 16hrs treatment, but the difference was not significant. In contrast, inhibition of calpain had no effect on both proteins, thereby indicating that the calpain system was not involved in a-SMN and FL-SMN degradation, at least in our experimental setting. To further verify the effect of MG132 treatment at the single cell level, particularly in terms of a-SMN or FL-SMN sub-cellular localization, we performed IF experiments in NSC34 cells co-transfected with Xpress-tagged FL-SMN and FLAG-tagged a-SMN. We used anti-Xpress or -FLAG antibodies to specifically recognize only the transfected (and not the endogenous) proteins. Before MG132 treatment, both FL-SMN and a-SMN were localized in cell bodies and neurites, with the granular (FL-SMN: Fig 4B_1_) or diffuse (a-SMN: Fig 4B_2_) staining pattern previously reported [13–14, 33]. MG132 incubation (8 hrs) deeply modified the morphology of treated NSC34 cells, which were characterized by shorter, thicker, irregular neurites with frequent neuritic swellings (Fig 4B_3_ and 4B_4_). FL-SMN was localized in coarser cytoplasmic granules, not extending into neurites (Fig 4B_3_), whereas a-SMN was diffusely distributed in the cell body and concentrated in the neuritic swellings (Fig 4B_4_). Morphological quantification confirmed that MG132 treatment induced a significant decrease of neuritic length (Fig 4C) and also revealed increased soma size of NSC34 co-transfected cells (Fig 4D). Furthermore, a statistically significant percentage of MG132 treated NSC34 cells showed FL-SMN localization restricted to the cell body (Fig 4E) and neuritic abnormalities (Fig 4F) when compared to untreated cells.

**Fig 5:**
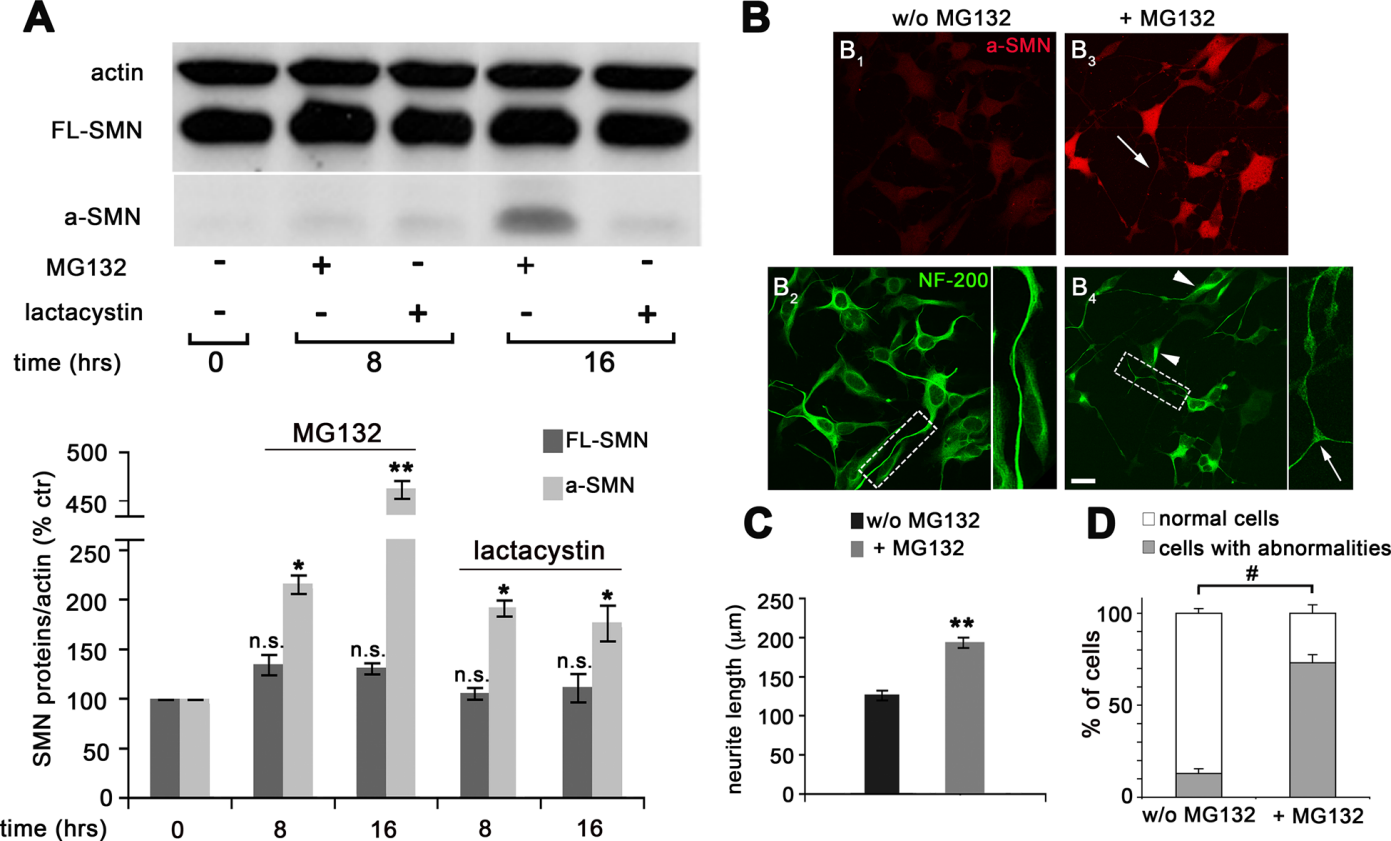
Effects of proteasome inhibitors on FL-SMN and a-SMN protein levels in a-SMN cell clones. **(A)** Western blot analysis and quantification of FL-SMN and a-SMN protein expression after MG132 or lactacystin treatment. The “+” and “-”indications represent the presence or absence, respectively, of proteasome inhibitor. The a-SMN81 cell clone was treated with MG132 or lactacystin for 8 or 16 hrs, and endogenous FL-SMN and a-SMN protein levels analyzed by Western blot with the anti-SMN antibody clone 8 recognizing the N-terminal region of both SMN proteins (upper panel). The a-SMN expression was significantly increased by both proteasome inhibitors (MG132: 8 hrs vs 0 hrs, *p<0.05; 16 hrs vs 0 hrs, **p<0.01; lactacystin: 8 and 16 hrs vs 0 hrs, *p<0.05). Conversely, not significant (n.s.) effect was present with either MG132 or lactacystin on FL-SMN protein levels at each time point vs untreated cells (0 hrs). Statistical analysis was performed by one-way ANOVA followed by Tukey HSD as post hoc comparison test. Distinct statistical tests were performed for the two protein datasets, i.e., one for a-SMN and one for FL-SMN, and statistical differences for each time points were reported vs untreated cells (0 hrs) in the graphs. (**B**) Representative confocal IF images (red: anti a-SMN in B_1_, B_3_; green: anti NF-200 in B_2_, B_4_) illustrating the a-SMN sub-cellular localization in the absence (B_1_-B_2_) or presence (B_3_-B_4_) of MG132. MG132 treated cells were characterized by slightly more branched neurites (B_4_, arrow in inset), neurofilament accumulation in the cell body and swellings along the neuritic shafts (Fig 5B_4_, arrowheads). Scale bar: 30 μm; 15 μm in insets. (**C**) Bar graphs showing the quantification of neurite length of a-SMN cell clones untreated and treated with MG132. Statistical analysis was performed by Student’s t-test (**p<0.01). (**D**) Stacked histograms showing the distribution of cell abnormalities in a-SMN cell clones untreated and treated with MG132. Data are presented as mean ± SEM of three different experiments. Percent ratio of cell abnormalities (normal cells/ cells with abnormalities) was compared by means of chi-square test (#p<0.001) between the two experimental conditions (w/o MG132 or + MG132).

Finally, we verified the data obtained after transient FL-SMN/a-SMN over-expression in NSC34 cell clones conditionally expressing a-SMN (clone a-SMN81) [33], i.e., in a cell system stably expressing detectable amounts of both SMN proteins. a-SMN81 cells grown in basal conditions were treated with both MG132 and lactacystin for 8 and 16 hrs (Fig 5). MG132 treatment significantly increased the steady-state levels of a-SMN, in a time-dependent fashion (Fig 5A). Lactacystin treatment was also effective in increasing a-SMN levels at both time points considered (Fig 5A). By contrast, neither MG132 nor lactacystin were able to influence the protein levels of endogenous FL-SMN in a-SMN expressing clones (Fig 5A). We also performed IF experiments to verify morphology and a-SMN sub-cellular localization after MG132 treatment in a-SMN clone81 (Fig 5B). IF analysis confirmed what observed at the WB level, i.e., the increase of a-SMN staining in MG132 treated cells (Fig 5B_1_–5B_3_). NF-200 stained MG132-treated clones were characterized by slightly more branched neurites (Fig 5B_4_, arrow in the inset), neurofilament accumulation in the cell bodies, and swellings along the neuritic shafts (Fig 5B_4_, arrowheads). For a better estimate of the morphologic abnormalities induced by MG132 treatment, we quantified neuritic length and the percentage of cells showing neuritic abnormalities. As indicated in Fig 5C and 5D, MG132 treatment significantly increased neuritic length (Fig 5C) and the percentage of cells with neuritic abnormalities (Fig 5D).

## Discussion

In this study, we analyze the intracellular fate of FL-SMN and a-SMN mRNAs and proteins to investigate the stability of both proteins and the pathways contributing to their degradation. Our data provided evidence that **i**) the stability of both FL-SMN and a-SMN transcripts was comparable; **ii**) the a-SMN protein was characterized by a much shorter half-life than FL-SMN; and **iii**) as already demonstrated for FL-SMN, the Ub/proteasome pathway played a major role in the a-SMN protein degradation.

The a-SMN transcript is generated by the retention of the short intron lying between exons 3 and 4 of the *SMN1* sequence, introducing a premature stop codon. Nonsense-mediated mRNA decay (NMD) is a general surveillance cell mechanism eliminating transcripts harboring a premature translation termination codon, including mutated mRNAs from SMA affected patients [35]. In theory, the low steady-state protein levels of a-SMN could be determined by the NMD mechanisms, affecting a-SMN mRNA stability and hence reducing the biological relevance of the protein. However, our data with both transcription or translation blockers clearly showed comparable stability of both a-SMN and FL-SMN transcripts at every time-point considered (Fig 2B and 2C). It is worth mentioning that similar mRNA stability was reported also for the FL-SMN and Δ7-SMN transcripts [36]. Based on the present data, no differential processing of the FL-SMN vs a-SMN transcripts, including NMD, could explain the different intracellular fate of the FL-SMN vs a-SMN proteins.

Other groups have previously analyzed the intracellular stability of FL-SMN vs Δ7-SMN proteins, showing that the two proteins were mainly degraded via the Ub/proteasome pathway and that the Δ7-SMN turnover was on average two-fold faster than that of FL-SMN [37–42]. In keeping with those previous results, the present data show that a-SMN is subjected to the same intracellular pathways degrading Δ7-SMN and FL-SMN. All three SMN isoforms have identical N-terminal structure and share in the protein N-terminus the majority of lysine residues, i.e., the potential canonical site for the start of the ubiquitination mechanisms.

According to the present data, the calpain system is unlikely to play a role in a-SMN degradation. Interestingly, neither a-SMN nor FL-SMN isoforms were substrates of the calpain degradation system in our experimental conditions. This is at variance with previous data demonstrating that FL-SMN is a direct target of calpain cleavage [40]. Even if most calpain cleavage sites were mainly located in the C-terminal part of FL-SMN sequence, i.e., outside the a-SMN sequence, a potent cleavage site was reported in exon 2b [41]. Therefore, we cannot exclude that the calpain cleavage of a-SMN might be relevant in more physiological in vivo conditions and not appreciable in our experimental settings.

More importantly, our data revealed that the a-SMN protein was characterized by a much shorter half-life than the FL-SMN counterpart. This was here clearly demonstrated in two different—neuronal and non-neuronal—cell settings after forced expression, and confirmed in a-SMN clones, i.e., in a cell setting stably expressing both SMN isoforms. Both FL-SMN and a-SMN proteins likely exert their effect more prominently during development. This is suggested by the a-SMN profile of expression on one side [13; 42; 43], and by recent results from different therapeutic strategies in mouse models for SMA. These latter data clearly demonstrated that the time-window for effective therapeutic intervention in SMA mice was restricted to the first post-natal days and that most likely no overt levels of the FL-SMN protein were required in the adult period [44]. The slower degradation rate of FL-SMN versus a-SMN is probably not related to the diverse function of the two proteins but rather to the protection provided by the protein complex in which the FL-SMN protein is assembled [45]. Indeed, the a-SMN and FL-SMN (and Δ7-SMN as well) differ for their C-terminal tail only. Most likely the diverse amino acid sequence of the C-terminus dictates different protein interactions and diverse protein complex formation that could explain the different localization and role in the neuronal compartment, and the lower expression and stability of the a-SMN protein. Previous studies demonstrated that deletion of FL-SMN exon 2 and 6 (i.e., domains relevant for FL-SMN self-association) decreased FL-SMN half-life, therefore indicating the relevance of oligomerization for FL-SMN stability [39]. Since the FL-SMN recruitment into a macromolecular complex made the protein more resistant to degradation than monomeric FL-SMN [39], it was proposed that FL-SMN existed in two biologic forms, the first with a relatively short life, the other assembled in a complex much more resistant to degradation. In addition, it was shown that direct interaction of FL-SMN with protein kinase ASK1 and the de-ubiquitinating enzyme Usp9x stabilized the protein that became less susceptible to proteasomal degradation [45–46]. The a-SMN protein retains the exon 2 but it lacks the exon 6 sequence. It is possible that a-SMN may interact less stably with other proteins through its different C-terminus and be therefore less protected from proteasome as well as other degradation systems. In this context, it is interesting to note (see Fig 3A and 3B; [13]) the possible cleavage of a-SMN at the C-terminus, further suggesting that a-SMN may be also degraded by other catabolic pathways when the proteasome system is blocked.

Since SMA is a typical loss-of-function disease and all SMN isoforms are preferentially degraded by the proteasome system, using proteasome inhibitors is theoretically a promising therapeutic option for human patients. In this regard, some drugs specifically affecting the proteasome system are FDA-approved and currently use in clinics (eg, Velcade for the treatment of multiple myeloma patients) [47]. Also, the proteasome inhibitor bortezomid significantly increased FL-SMN protein levels and improved motor function of SMA mice [45]. However, general inhibition of proteasome activity could be very toxic in the long-term setting of the treatment of a neurodegenerative disease. In addition, our morphologic and morphometric analysis of cells treated with proteasome inhibitors did not seem to exert beneficial effects. Even if MG132 treatment increased neuritic length in a-SMN cell clones (Fig 5C), **i**) the absence of the normal FL-SMN trafficking into neurites of NSC34 co-transfected cells (Fig 4E), **ii**) the a-SMN localization in abnormal neurite regions of a-SMN clones where neurofilament accumulation was evident (Fig 5B–5D), as well as **iii**) the decreased neuritic length, increased soma size, and overt neuritic abnormalities of NSC34 co-transfected cells (Fig 4C, 4D–4F) are all factors indicating MG132-induced alterations of the normal axonal growth or homeostasis. Obviously, these data need to be interpreted very cautiously, because blocking the proteasome could easily interfere with most protein systems, reasonably affecting the homeostasis of the cell independently from the SMN system itself.

## References

[1] Pearn J. Classification of spinal muscular atrophies. Lancet 1980; 1:919–922. 6103267 10.1016/s0140-6736(80)90847-8

[2] Sun Y, Grimmler M, Schwarzer V, Schoenen F, Fischer U, Wirth B. Molecular and functional analysis of intragenic SMN1 mutations in patients with spinal muscular atrophy. Hum Mutat. 2005; 25: 64–71. 15580564 10.1002/humu.20111

[3] Prior TW, Snyder PJ, Rink BD, Pearl DK, Pyatt RE, Mihal DC, et al Newborn and carrier screening for spinal muscular atrophy. Am J Med Genet. 2010; 152: 1608–16. 10.1002/ajmg.a.33474 20578137

[4] Lefebvre S, Burglen L, Reboullet S, Clermont O, Burlet P, Viollet L, et al Identification and characterization of a spinal muscular atrophy-determining gene. Cell 1995; 80: 155–165. 7813012 10.1016/0092-8674(95)90460-3

[5] Gennarelli M, Lucarelli M, Capon F, Pizzuti A, Merlini L, Angelini C, et al Survival motor neuron gene transcript analysis in muscles from spinal muscular atrophy patients. Biochem. Biophys. Res Commun. 1995; 213: 342–348. 7639755 10.1006/bbrc.1995.2135

[6] Campbell L, Potter A, Ignatius J, Dubowitz V, Davies K. Genomic variation and gene conversion in spinal muscular atrophy: implications for disease process and clinical phenotype. Am J Hum Genet. 1997; 61: 40–50. 9245983 10.1086/513886 PMC1715870

[7] Coovert DD, Le TT, Mcandrew PE, Strasswimmer J, Crawford TO, Mendell JR, et al The survival motor neuron protein in spinal muscular atrophy. Hum Mol Genet. 1997; 6: 1205–1214. 9259265 10.1093/hmg/6.8.1205

[8] Mcandrew PE, Parsons DW, Simard LR, Rochette C, Ray PN, Mendell JR, et al Identification of proximal spinal muscular atrophy carriers and patients by analysis of SMNT and SMNC gene copy number. Am J Hum Genet. 1997; 60: 1411–1422. 9199562 10.1086/515465 PMC1716150

[9] Monani UR, Lorson CL, Parsons DW, Prior TW, Androphy EJ, Burghes AH, et al A single nucleotide difference that alters splicing patterns distinguishes the SMA gene SMN1 from the copy gene SMN2. Hum Mol Genet. 1999; 8: 1177–1183. 10369862 10.1093/hmg/8.7.1177

[10] Lorson CL, Androphy EJ. An exonic enhancer is required for inclusion of an essential exon in the SMA-determining gene SMN. Hum Mol Genet. 2000; 9: 259–65. 10607836 10.1093/hmg/9.2.259

[11] Vitali T, Sossi V, Tiziano F, Zappata S, Giuli A, Paravatou-Petsotas M, et al Detection of the survival motor neuron [SMN] genes by FISH: further evidence for a role for SMN2 in the modulation of disease severity in SMA patients. Hum Mol Genet. 1999; 8: 2525–2532. 10556301 10.1093/hmg/8.13.2525

[12] Wirth B, Brichta L, Hahnen E. Spinal muscular atrophy: from gene to therapy. Semin Pediatr Neurol 2006; 13: 121–131. 17027862 10.1016/j.spen.2006.06.008

[13] Setola V, Terao M, Locatelli D, Bassanini S, Garattini E, Battaglia G. Axonal-SMN [a-SMN], a protein isoform of the survival motor neuron gene, is specifically involved in axonogenesis. Proc Natl Acad Sci U S A 2007; 104: 1959–1964. 17261814 10.1073/pnas.0610660104 PMC1794299

[14] Locatelli D, d'Errico P, Capra S, Finardi A, Colciaghi F, Setola V, et al Spinal muscular atrophy pathogenic mutations impair the axonogenic properties of axonal-survival of motor neuron. J Neurochem 2012a; 121: 465–74. 22324632 10.1111/j.1471-4159.2012.07689.x

[15] Liu Q, Dreyfuss G. A novel nuclear structure containing the survival of motor neurons protein. EMBO J. 1996; 15: 3555–65. 8670859 PMC451956

[16] Meister G, Buhler D, Pillai R, Lottspeich F, Fischer U. A multiprotein complex mediates the ATP-dependent assembly of spliceosomal U snRNPs. Nat Cell Biol. 2001; 3: 945–949. 11715014 10.1038/ncb1101-945

[17] Pellizzoni L, Yong J, Dreyfuss G. Essential role for the SMN complex in the specificity of snRNP assembly. Science 2002; 298: 1775–9. 12459587 10.1126/science.1074962

[18] Gubitz AK, Feng W, Dreyfuss G. The SMN complex. Exp Cell Res. 2004; 296: 51–56. 15120993 10.1016/j.yexcr.2004.03.022

[19] Wan L, Battle DJ, Yong J, Gubitz AK, Kolb SJ, Wang J, et al The survival of motor neurons protein determines the capacity for snRNP assembly: biochemical deficiency in spinal muscular atrophy. Mol Cell Biol. 2005; 25: 5543–51. 15964810 10.1128/MCB.25.13.5543-5551.2005 PMC1156985

[20] Carissimi C, Saieva L, Gabanella F, Pellizzoni L. Gemin8 is required for the architecture and function of the survival motor neuron complex. J Biol Chem. 2006; 281: 37009–16. 17023415 10.1074/jbc.M607505200

[21] Zhang Z, Lotti F, Dittmar K, Younis I, Wan L, Kasim M, et al SMN deficiency causes tissue-specific perturbations in the repertoire of snRNAs and widespread defects in splicing. Cell. 2008; 133: 585–600 10.1016/j.cell.2008.03.031 18485868 PMC2446403

[22] Bäumer D, Lee S, Nicholson G, Davies JL, Parkinson NJ, Murray LM, et al Alternative splicing events are a late feature of pathology in a mouse model of spinal muscular atrophy. PLoS Genet. 2009; 5(12):e1000773 10.1371/journal.pgen.1000773 20019802 PMC2787017

[23] Sleigh JN, Gillingwater TH, Talbot K. The contribution of mouse models to understanding the pathogenesis of spinal muscular atrophy. Dis Model Mech. 2011; 4: 457–67. 10.1242/dmm.007245 21708901 PMC3124050

[24] Burghes AH, Beattie CE. Spinal muscular atrophy: why do low levels of survival motor neuron protein make motor neurons sick? Nat Rev Neurosci. 2009; 10:5 97–609. 10.1038/nrn2670 PMC2853768 19584893

[25] Battaglia G, Princivalle A, Forti F, Lizier C, Zeviani M. Expression of the SMN gene, the spinal muscular atrophy determining gene, in the mammalian central nervous system. Hum Mol Genet. 1997; 6: 1961–1971. 9302277 10.1093/hmg/6.11.1961

[26] Pagliardini S, Giavazzi A, Setola V, Lizier C, Di Luca M, DeBiasi S, et al Subcellular localization and axonal transport of the survival motor neuron [SMN] protein in the developing rat spinal cord. Hum Mol Genet 2000; 9: 47–56. 10587577 10.1093/hmg/9.1.47

[27] Jablonka S, Bandilla M, Wiese S, Bühler D, Wirth B, Sendtner M, et al Co-regulation of survival of motor neuron [SMN] protein and its interactor SIP1 during development and in spinal muscular atrophy. Hum Mol Genet. 2001; 10: 497–505. 11181573 10.1093/hmg/10.5.497

[28] Rossoll W, Jablonka S, Andreassi C, Kroning AK, Karle K, Monani UR, et al Smn, the spinal muscular atrophy-determining gene product, modulates axon growth and localization of beta-actin mRNA in growth cones of motoneurons. J Cell Biol. 2003; 163: 801–812. 14623865 10.1083/jcb.200304128 PMC2173668

[29] Todd AG, Morse R, Shaw DJ, McGinley S, Stebbings H, Young PJ. SMN, Gemin2 and Gemin3 associate with beta-actin mRNA in the cytoplasm of neuronal cells in vitro. J Mol Biol. 2010; 40: 681–9. 10.1016/j.jmb.2010.06.058 20620147

[30] Fallini C, Zhang H, Su Y, Silani V, Singer RH, Rossoll W, et al The survival of motor neuron [SMN] protein interacts with the mRNA-binding protein HuD and regulates localization of poly[A] mRNA in primary motor neuron axons. J Neurosci. 2011; 31: 3914–25. 10.1523/JNEUROSCI.3631-10.2011 21389246 PMC3070748

[31] Akten B, Kye MJ, Hao le T, Wertz MH, Singh S, Nie D, et al Interaction of survival of motor neuron [SMN] and HuD proteins with mRNA cpg15 rescues motor neuron axonal deficits. Proc Natl Acad Sci U S A. 2011; 108: 10337–42. 10.1073/pnas.1104928108 21652774 PMC3121858

[32] Yamada H, Nishida Y, Maihara T, Sa'adah N, Harahap NI, Nurputra DK, Ar Rochmah M, Nishimura N, Saito T, Kubo Y, Saito K, Nishio H. Two Japanese Patients With SMA Type 1 Suggest that Axonal-SMN May Not Modify the Disease Severity. Pediatr Neurol. 2015; 52: 638–41. 10.1016/j.pediatrneurol.2015.02.023 25838041

[33] Locatelli D, Terao M, Fratelli M, Zanetti A, Kurosaki M, Lupi M, et al Human axonal survival of motor neuron [a-SMN] protein stimulates axon growth, cell motility, C-C motif ligand 2 [CCL2], and insulin-like growth factor-1 [IGF1] production. J Biol Chem. 2012b; 287: 25782–94. 22669976 10.1074/jbc.M112.362830 PMC3406665

[34] Cashman NR, Durham HD, Blusztajn JK, Oda K, Tabira T, Shaw IT, et al Neuroblastoma x spinal cord [NSC] hybrid cell lines resemble developing motor neurons. Dev Dyn. 1992; 194: 209–221. 1467557 10.1002/aja.1001940306

[35] Brichta L, Garbes L, Jedrzejowska M, Grellscheid SN, Holker I, Zimmermann K, et al Nonsense-mediated messenger RNA decay of survival motor neuron 1 causes spinal muscular atrophy. Hum Genet. 2008; 123: 141–53. 10.1007/s00439-007-0455-7 18172693

[36] Heier CR, Gogliotti RG, DiDonato CJ. SMN transcript stability: could modulation of messenger RNA degradation provide a novel therapy for spinal muscular atrophy? J Child Neurol. 2007; 22: 1013–8. 17761657 10.1177/0883073807305669

[37] Chang HC, Hung WC, Chuang YJ, Jong YJ. Degradation of survival motor neuron [SMN] protein is mediated via the ubiquitin/proteasome pathway. Neurochem Int. 2004; 45: 1107–12. 15337310 10.1016/j.neuint.2004.04.005

[38] Vitte J, Fassier C, Tiziano FD, Dalard C, Soave S, Roblot N, et al Refined characterization of the expression and stability of the SMN gene products. Am J Pathol. 2007; 171: 1269–80. 17717146 10.2353/ajpath.2007.070399 PMC1988876

[39] Burnett BG, Munoz E, Tandon A, Kwon DY, Sumner CJ, Fischbeck KH. Regulation of SMN protein stability. Mol Cell Biol. 2009; 29: 1107–1115. 10.1128/MCB.01262-08 19103745 PMC2643817

[40] Walker MP, Rajendra TK, Saieva L, Fuentes JL, Pellizzoni L, Matera AG. SMN complex localizes to the sarcomeric Z-disc and is a proteolytic target of calpain. Hum Mol Genet. 2008; 17: 3399–410. 10.1093/hmg/ddn234 18689355 PMC2566527

[41] Fuentes JL, Strayer MS, Matera AG. Molecular determinants of survival motor neuron [SMN] protein cleavage by the calcium-activated protease, calpain. PLoS One 2010; 5:e15769 10.1371/journal.pone.0015769 21209906 PMC3012718

[42] Burlet P, Huber C, Bertrandy S, Ludosky MA, Zwaenepoel I, Clermont O, et al The distribution of SMN protein complex in human fetal tissues and its alteration in spinal muscular atrophy. Hum Mol Genet. 1998; 7: 1927–33. 9811937 10.1093/hmg/7.12.1927

[43] Gabanella F, Carissimi C, Usiello A, Pellizzoni L. The activity of the spinal muscular atrophy protein is regulated during development and cellular differentiation. Hum Mol Genet. 2005; 14: 3629–42. 16236758 10.1093/hmg/ddi390

[44] Robbins KL, Glascock JJ, Osman EY, Miller MR, Lorson CL. Defining the therapeutic window in a severe animal model of spinal muscular atrophy. Hum Mol Genet. 2014; 23: 4559–68. 10.1093/hmg/ddu169 24722206 PMC4119406

[45] Kwon JE, Kim EK, Choi EJ. Stabilization of the survival motor neuron protein by ASK1. FEBS Lett. 2011; 585: 1287–92. 10.1016/j.febslet.2011.04.011 21496457

[46] Han KJ, Foster DG, Zhang NY, Kanisha K, Dzieciatkowska M, Sclafani RA, et al Ubiquitin-specific protease 9x deubiquitinates and stabilizes the spinal muscular atrophy protein-survival motor neuron. J Biol Chem. 2012; 287: 43741–52. 10.1074/jbc.M112.372318 23112048 PMC3527959

[47] Twombly R. First proteasome inhibitor approved for multiple myeloma. J Natl Cancer Inst. 2003; 95:845 12813164 10.1093/jnci/95.12.845

